# Supporting Self-Regulated Learning in Medical School: A National Survey

**DOI:** 10.7759/cureus.108984

**Published:** 2026-05-16

**Authors:** Anna Romanova, Susan Humphrey-Murto, Craig Campbell, Douglas Archibald, Sydney Ruller, Lina Shoppoff, Claire Touchie

**Affiliations:** 1 Department of Medicine, Division of General Internal Medicine, The Ottawa Hospital, Ottawa, CAN; 2 Department of Medicine, Division of Rheumatology, The Ottawa Hospital, Ottawa, CAN; 3 Departments of Family Medicine, Innovation in Medical Education, and Faculty of Education, University of Ottawa, Ottawa, CAN; 4 Department of Medicine, The Ottawa Hospital, Ottawa, CAN; 5 Department of Family Medicine, The Ottawa Hospital, Ottawa, CAN

**Keywords:** goal setting, learning plans, lifelong learning, self-regulated learning, undergraduate medical education

## Abstract

Introduction

Physicians must continuously update their knowledge and skills to maintain competency, yet the self-regulated learning (SRL) skills required for lifelong learning are not innate and must be learned from the start of medical training. Learning plans (LPs) are an effective SRL support strategy but are inconsistently used in undergraduate medical education (UME). How best to support lifelong learning for medical students and sustain it after graduation remains unclear.

Methods

A national cross-sectional survey of 17 Canadian UME programs was conducted between November 2023 and January 2024 to determine how they are supporting SRL skill development, whether they use LPs to do so, and identify barriers and facilitators to LP use. Data were analysed using descriptive and qualitative content analyses.

Results

Of the total, 10 of 17 (59%) UME programs responded to the survey. Schools use different terminology when talking about SRL and approach SRL and LP implementation in variable ways. Eight schools (80%) currently use LPs, mainly for struggling students. Barriers to LP use for students and faculty include a lack of SRL skills, LP development training, time, engagement, guidance, and feedback. Facilitators include SRL skills training, using a framework for goal setting and LP development, as well as co-creation with faculty.

Conclusion

Canadian UME programs demonstrate a diversity in approaches and strategies for SRL support. However, standardized evidence-based practices are needed to enhance SRL skill development for both students and faculty and optimize the use of LPs, not just within the context of remediation but as a proactive tool for lifelong learning success for all.

## Introduction

Driven by advancements such as artificial intelligence and emerging technologies, the practice of medicine is rapidly evolving. Physicians must continuously update their knowledge and skills to sustain and enhance their competence. This ongoing need underscores the importance of self-regulated learning (SRL), a competency that enables active, lifelong learning. Developing SRL skills early in training is essential, which the Association of Faculties of Medicine of Canada (AFMC) has recognized as a key priority within the accreditation standards for undergraduate medical education (UME) [1]. These skills are not innate, and medical students must learn to set, plan, execute, monitor, and reflect on their learning goals in a structured and intentional way to effectively engage in lifelong learning. However, standardized evidence-based guidance on how to best support SRL skill development in UME is currently lacking.

SRL theory explains how learners regulate their learning and has been defined as a cyclical process where learners actively control their thoughts, actions, and motivation to achieve their goals [2]. Several models of SRL exist, but all entail that the learner is responsible for setting, planning, executing, monitoring, and reflecting on their learning goals [2]. Goal setting is a key component in SRL, and goal-setting theory suggests that specific and difficult goals lead to higher performance, increased internal motivation, and satisfaction [3]. Commitment to goals, goal importance, self-efficacy, feedback on goal progress, and satisfaction when goals are achieved are all important factors that affect performance [3]. However, simply creating goals is not sufficient to achieve the intended outcomes. Learners need to generate a detailed plan and take an active role in its implementation and evaluation.

Medical students require support across all stages of goal setting and SRL, largely due to their limited experience with these skills [4]. Like other learners, they often struggle with accurate self-assessment and may overlook areas of weakness without appropriate guidance [5,6]. Consequently, they face challenges in independently identifying meaningful learning goals and in determining how to effectively pursue them [7]. Interpreting and contextualizing feedback can also be difficult without external support, limiting their ability to translate feedback into actionable improvement [8,9]. These challenges are further amplified during the transition from classroom-based to workplace-based learning, where the complexity and pace of clinical environments can hinder students’ ability to self-regulate effectively [10].

Learning plans (LPs) are an educational tool grounded in SRL and goal-setting theories that allow learners to take an active role in their education [11]. By requiring the learner to self-identify what they need to learn and why, how they are going to do it, how they will know when they have accomplished their learning, define the timeframe for goal achievement, and assess the impact of their learning, LPs are well suited to support lifelong learning and the development of SRL skills for all learners, not just those in academic difficulty [11,12]. However, in medical education, most of the experience with LP use lies in postgraduate training, with some residency training programs in Canada and the US recently introducing mandatory LPs [13,14]. A recent scoping review on LP use in UME found that LPs have not been consistently used in UME, with no common framework for their development, implementation, or assessment, and no Canadian studies have been found [12]. While adoption of LPs at the UME stage may facilitate the translation of SRL skills into residency training and beyond, how best to support lifelong learning for medical students remains unclear.

Aims and objectives

Given this gap in the literature, the primary aim of this study was to survey Canadian UME programs to determine how they are supporting SRL skill development for their students. Specific study objectives were to: (1) elicit current strategies or tools being used to promote SRL skill development; (2) determine whether LPs are being used as a tool for SRL skill development; (3) if LPs are being used: (a) determine how they are used; (b) identify perceived barriers and facilitators to their use.

## Materials and methods

A cross-sectional survey was distributed electronically to 17 Canadian UME programs between November 2023 and January 2024. This study was approved by the Ottawa Health Science Network Research Ethics Board (ID 20230330-01H).

Survey development

Survey development followed Phillips et al.’s [15] six-step approach for survey design (needs assessment, survey construction, establishing evidence, survey delivery, data analysis, reporting guidelines). Survey design was guided by goal-setting and SRL theories [2,3], findings from the recent scoping review on LP use in UME [12], and a literature search on LP use in other health professions education (HPE) programs. In addition to using theoretical frameworks to guide survey construction and data analysis, content validity was further enhanced through the involvement of medical education experts in our study team, who reviewed the preliminary survey to ensure it adequately covered the relevant aspects of the concept being measured. Next, we piloted the survey with eight physicians from various specialties participating in a yearlong Healthcare Education Scholars Program at the University of Ottawa. Based on their feedback, the questionnaire was refined to improve item clarity, response options, and survey flow. Definitions of key concepts like SRL skills, as well as examples of LP development, monitoring, and assessment frameworks, were added. Item order was adjusted to improve logical flow and enhance ease of completion. Finally, the online survey was pre-tested by the whole study team to ensure technical functionality prior to dissemination to the study population.

The final survey instrument is provided in Appendix A and consists of five domains. The first domain includes two items to collect generic information about the individual medical school’s SRL support strategies using mandatory free-text box responses. The second domain consists of 12 items to explore their experience with LPs using “Yes” or “No” questions with mandatory free-text boxes to elaborate on any “Yes” responses. This domain was developed based on the core components of LPs identified in the literature [11,12]. The third and fourth domains entail 19 and 18 items, respectively, to determine the presence and strength of perceived barriers and facilitators to LP use in UME for students and faculty using four-point Likert scales. This domain was structured according to barriers and facilitators to LP use in UME identified in the literature [12], as well as goal-setting and SRL best practices [2,3]. The final domain consists of 15 items to assess the presence of current or future SRL-promoting strategies or tools based on goal-setting and SRL theories [2,3]. Non-mandatory free-text space was provided at the end of each domain to facilitate additional information or elaboration on respondents’ selections. Skip logic was applied at the beginning of the second domain to allow participants to skip the second, third, and fourth domains if their school was not currently using LPs.

Participants

Since survey items focused primarily on institutional practices rather than individual opinions, one primary contact (UME dean, assistant dean, or vice dean) was identified at each of the 17 Canadian medical schools by AK. These contacts were chosen as they would be the most knowledgeable about their school’s UME curriculum and, therefore, best placed to provide accurate and in-depth responses. Demographic data for each medical school were collected from their websites (accessed March 2024) and the 2021 AFMC Canadian Medical Education Statistics (CMES) report [16].

Data collection

Primary contacts at each medical school were invited to participate in the survey via personalized emails. The survey was delivered via REDCap (v13.7.20, 2024 Vanderbilt University), a secure online survey platform, and responses were collected anonymously. The survey was translated into French for distribution to the four Francophone medical training programs. The survey was open for two weeks, and an email reminder was sent to non-responders at the halfway point, as well as one day before it closed. Participants who completed the survey were offered a $40 gift card.

Data analysis

The long survey period, reminder emails, and completion incentive were used to reduce nonresponse bias. Data from REDCap was exported into Excel (Microsoft® Corp., Redmond, WA, USA). Quantitative data were analysed using descriptive statistics (mean, percentage). Missing data were excluded from the analysis. Open-ended responses were treated as an adjunct analysis to better understand the primary quantitative survey questions [17]. Qualitative content analysis was used to analyse these open-ended survey responses in a primarily deductive fashion due to the nature of the questionnaire; however, the authors remained open to the identification of any unexpected themes [18]. Since the survey questions were primarily of a more pragmatic nature, a manifest analysis was performed, whereby the investigators described what is visible and obvious in the text and did not seek to extend understanding to the interpretive level [19]. CT and SHM independently read all the qualitative data and inductively developed initial codes. Over several meetings with the full research team, codes were reviewed and refined, and themes were developed through consensus.

## Results

Of the total, 10 of the 17 (59%) Canadian medical schools responded to the survey. Three (30%) were from Western Canada, five (50%) from Ontario, one (10%) from Quebec, and one (10%) from Atlantic Canada. One (10%) school was Francophone, and one (10%) was bilingual; the rest were Anglophone (n = 8, 80%). UME programs at most schools were four years in duration (n = 8, 80%), three years at one school (10%), and up to five years at one school (10%). All schools followed the traditional UME curriculum, consisting of pre-clinical (pre-clerkship) followed by clinical (clerkship) training.

Current SRL support structures

Responding schools educated students on SRL skills (n = 5, 50%), goal setting (n = 7, 70%), strategies to achieve learning goals (n = 8, 80%), and self-reflection skills to assess the impact of their learning (n = 10, 100%). Faculty were similarly educated on SRL skills (n = 4, 40%), goal setting (n = 5, 50%), strategies to help students achieve their learning goals (n = 4, 40%), self-reflection skills to help students assess the impact of their learning (n = 5, 50%), and effective feedback skills (n = 9, 90%). Three (30%) schools required a specific learning goal structure, like the SMART template (specific, measurable, achievable, relevant, time-bound) [20], and three (30%) schools used a learning goal documentation system to monitor progress on goal achievement. Some students had regularly scheduled time to create learning goals (n = 4, 40%) and follow up on their progress (n = 3, 30%). Some faculty mentors were required to assist students with learning goal creation (n = 3, 30%) and follow up on their progress (n = 3, 30%).

Qualitative analysis of open-text responses (Table 1) showed that schools use different terminology and approaches when talking about SRL, with terms like independent study, self-directed learning, and self-learning used. They also approach SRL skill development in variable ways. Strategies include introductory modules on self-directed learning, dedicated time for independent study, provision of study tips and resources, facilitated small- and large-group sessions to reflect on learning, problem-based and case-based learning sessions, frequent low-stakes programmatic assessments and feedback, and faculty-guided portfolio sessions. At some schools, this is an explicit curricular objective that is addressed throughout the duration of medical school or as a formal course in which students create their own learning goals and plans to develop self-directed scholarly projects.

**Table 1:** Identified themes related to self-regulated learning (SRL) skill development and learning plan (LP) use in Canadian undergraduate medical education (UME) programs. The bolded words in this table highlight key terms and phrases related to the theme or sub-theme in each row.

Themes	Sub-themes	Illustrative quotes
Terminology variability	Schools use different terminology when talking about SRL	(i) “Students in pre-clerkship have designated time in the timetable for independent study.” (ii) “…we use the term self-directed learning in our program.” (iii) “Faculty-guided portfolio session and student-led CBL session…help promote SRL.”
Approach variability	Schools approach SRL and LP implementation in variable ways	(i) “The program includes a podcast on self-regulation and then a mentor helps the students identify areas for improvement, create learning goals, and then reflect on their progress over time.” (ii) “Our students create their own learning goals and plans as they relate to UGME exit competencies and the CanMEDS roles.” (iii) “It is an explicit curricular objective, that is addressed in multiple courses across the 4 years.”
Support for academic difficulty	Schools use LPs mainly for struggling students	(i) “For students who are underperforming on clerkship courses, we enroll them in a mentorship-based remediation program that helps them with their approach to learning and self-regulation.” (ii) “Learning plans are used with students who are experiencing academic difficulty or who are on leaves from the program and continuing to engage in self-directed learning during that time.”

Current LP use

LPs were mentioned on one school’s website (10%) as part of a portfolio throughout all four years of medical school. From survey responses, eight schools (80%) currently use LPs, and one (10%) is planning to introduce them. LPs tend to be used with pre-clinical students and in a variety of ways: as part of remediation for struggling learners (n = 7, 88%), as a mandatory longitudinal activity (n = 1, 12%), or as an elective activity in which any student can take part (n = 1, 12%).

Two of the schools that use LPs for remediation did not provide further details on their use. LPs are developed by the student with a mentor at five schools (83%) and by the mentor alone at one school (17%). Two schools (33%) use a framework for LP development, such as SMART goals or CanMEDS competencies [20,21]. At two schools (33%), LP quality is assessed by the mentor before it is implemented, one by the SMART framework, while no details were provided for the other school. Two of the six schools (33%) provide training for students on LP development and use, while three schools (50%) do the same for faculty. Four schools (67%) monitor LP progress, and at one school (17%), LPs are assessed by faculty members with written narrative feedback using a rubric looking at three domains: self-ownership of learning, integrating scholarship, and connecting learning to the role of the scholar.

Barriers and facilitators to LP use

Data on barriers and facilitators to LP use is available for six of the eight schools (75%) that reported currently using LPs. Two of these schools (33%) responded “don’t know” for most items. Nevertheless, general themes were identified from the remaining four (67%) respondents.

Students were perceived to have difficulty with SRL skills to identify learning goals, create effective plans, and achieve them. Barriers to LP use included lack of time to create them, engagement with the process and commitment to complete them, opportunities to achieve learning goals, and feedback on goal progress. Additionally, faculty were perceived to lack the skills required to help students develop learning goals, time to guide and review LPs with students, and engagement with the process.

Perceived facilitators for students included receiving teaching on LPs and goal-setting, using a framework for goal setting and LP development, having protected time to create and review their LPs with a mentor, and using a documentation system to monitor their progress. Faculty guidance and feedback to set learning goals, create and implement LPs, and monitor progress were important.

## Discussion

This study found that 10 Canadian medical schools approach SRL and LP implementation in variable ways. The qualitative data illustrated that there is no unified language defining SRL across the responding schools, and there is also no standardized practice to promote lifelong learning. While many elements of goal-setting and SRL theories were reflected in the schools’ support strategies, few currently offer explicit full SRL cycle training for trainees or faculty on how to plan, execute, monitor, and reflect on students’ learning goals in a structured way. While LPs are just one tool that can be used to support SRL skill development, most schools (80%) reported currently using them, but mainly with struggling students to structure a clear remediation plan. Perceived barriers and facilitators to LP use relate to all stages of goal setting and the SRL cycle, as well as LP development, execution, and monitoring.

An important finding is that current support strategies to develop SRL skills tend to focus on training students, with less focus on faculty development. This contradicts what we know about how medical students self-regulate their learning and their need for guidance at all stages of goal setting and SRL [4-10,22]. Co-creation with an experienced mentor and supervisor engagement throughout LP development and implementation have also been identified as key facilitating factors for LP use [12,23]. Moreover, faculty involvement and training are vital to sustaining curricular change [24], so any SRL support strategies would need to target both students and faculty to be most effective.

Results from this study complement findings from the recent scoping review on LP use in UME, where most of the 39 included studies were pilot initiatives with no common framework for LP development, implementation, or assessment [12]. However, LPs appeared well-suited to support workplace learning for medical students, as they facilitated the development of SRL skills, including improved self-assessment and goal-setting skills [25,26], goal attainment, especially when guided by a mentor [27-30], and improved feedback [31]. In many of the studies, SRL skill development strategies were similarly directed mostly at enhancing trainees’ skills. Finally, similar barriers and facilitators to LP use were identified for both students and faculty. For medical students, perceived lack of SRL skills was identified as the biggest barrier, which is also seen with other HPE students, including those in postgraduate medicine [32,33], nursing [34,35], respiratory therapy [36], physiotherapy [37,38], and occupational therapy [39]. However, in contrast to our survey findings, only six (15%) of the studies in the scoping review used LPs specifically for low-performing students [40-45], suggesting the benefits described above apply to all learners.

The descriptive nature of this study limits our ability to draw causal conclusions about the survey findings; however, a few possibilities can be explored. The lack of conceptual consistency about SRL and the lack of formal guidance on how to best support SRL skill development likely contribute to the diversity of practice currently seen in UME. Longitudinal SRL skill development and LP implementation for all students would also require significant human resources, time investment, and organizational support. Medical training programs have limited resources and need to prioritize their allocation across multiple competing demands, which likely also accounts for the variability in SRL support strategies being implemented, as well as why LPs remain used mainly for struggling learners despite evidence supporting their broader use.

Limitations

While our survey response rate was over 50%, the overall sample size was still small, and the results mainly reflect data from Ontario and Western Canada. Only one of the four schools from Quebec and one of the two schools from Atlantic Canada responded to our survey, limiting the generalizability of the results and potentially missing unique perspectives from those regions. Other than three of the seven non-responding schools being Francophone, no systematic difference between responding and non-responding schools was apparent. Our findings are also specific to the Canadian context, limiting transferability to other settings; however, we intentionally focused on Canadian UME programs given the absence of literature in this specific context. Survey findings may also be affected by recall and social desirability biases inherent to self-reported data. While the use of a single key informant per institution was intentional, including more than one respondent per school may have improved data reliability and validity by allowing for response cross-validation and consensus assessment. Finally, the survey format inherently limits the depth of understanding that can be obtained about the topic of study, and the descriptive study design limits the ability to draw causal conclusions from the findings.

Future directions

Future research is required to explore the most effective way to support the development and assessment of SRL skills for both medical students and faculty. Unified language and data-driven guidance would help medical schools consolidate their resources to implement evidence-based strategies that support SRL skill development. Given the demonstrated benefits of LPs in supporting SRL skill development, studies to determine a standardized framework for LP development, implementation, and assessment are needed to guide their use for all students, not just those in academic difficulty. Finally, future studies can investigate whether and how SRL skills can be sustained across the education continuum from UME to graduate medical education (GME) and beyond to independent practice. For example, there is currently very limited data on the long-term impact of LPs, and future work can assess whether enhanced SRL skills, as well as academic and clinical performance resulting from LP use, are sustained over time.

## Conclusions

Development of lifelong learning skills is a priority for Canadian UME programs, with a multitude of approaches currently being used to support SRL. LPs are one of those support strategies, with evidence that they can help all learners; however, in Canada, they are used mostly in the context of remediation. Key barriers and facilitators to LP use were identified, including training on SRL skills and LP development, using a framework for goal setting and LP development, ensuring protected time for LP creation and monitoring, and co-creating LPs with faculty guidance and feedback. Ultimately, there is a need to establish standardized, evidence-based practices to streamline medical school resource use, enhance SRL skill development for both students and faculty, and optimize the use of LPs, not just within the context of remediation but as a proactive tool for lifelong learning success for all.

## Appendices

Appendix A: Complete survey

Supporting Self-Regulated Learning in Medical School: A National Survey

Please select your medical school (this response is for tracking purposes only and will not be linked to the rest of your survey responses):

Drop-down menu of 17 medical faculties

Section A - Self-Regulated Learning Strategies and Tools

In order to engage in lifelong learning, students must develop self-regulated learning (SRL) skills. SRL entails that the trainee is responsible for setting, planning, executing, monitoring, and reflecting on their learning goals.

1. How does your school currently support SRL skill development for your students? (you will have an opportunity to describe future strategies in the next question (mandatory free text box to answer)

2. What additional strategies or tools are in development for implementation at your school in the next two years? Please describe. (mandatory free text box to answer)

Section B - Learning Plans

Learning plans (LPs) (also known as learning contracts, personal development plans, or personal action plans) are an educational tool that allows learners to take an active role in their training and, therefore, may be well suited to support SRL. In this section, you will be asked to share your experience with LP development and implementation at your school.

1. Are LPs used in your medical school?

Yes

No

Planning to introduce them

*if No - 2. Please describe why LPs are not used in your medical school (mandatory free text box)

*skip logic - if No or planning to introduce them, go to Section E after answering Q2 above

2. Which students use LPs? Select all that apply:

- Year 1

- Year 2

- Year 3

- Year 4

- Other (please describe):

3. Does your school use a framework for LP development? ex: SMART, CanMEDS

Yes

No

Don’t know

*if Yes - 3a. Please describe the framework (mandatory free text box)

4. Who develops the LP?

Student alone

Student with mentor

Mentor alone

5. Is the LP assessed for quality prior to implementation? ex: SMART

Yes

No

Don’t know

*if Yes - 5a. Please describe the quality assessment tool (mandatory free text box)

6. In what context are LPs used in your medical school? Select all that apply:

- Mandatory, part of longitudinal curriculum (ie, outside of clinical rotations)

- Mandatory, part of the individual rotation’s curriculum

- Mandatory remediation of struggling learners 

- Elective activity that any students can do if they want

- Other (please describe):

*if part of rotation - 6a. Which rotation(s) use LPs? (mandatory free text box)

7. Is there training for students on LP development and use?

Yes

No

*if Yes - 7a. Please describe the training (mandatory free text box)

8. Is there training for faculty on LP development and use?

Yes

N

*if Yes - 8a. Please describe the training (mandatory free text box)

9. Is progress on LP monitored? ex: assess goal achievement half-way through LP timeline

Yes

No

*if Yes - 9a. Please describe how it is monitored (mandatory free text box)

10. Are LPs graded or assessed?

Yes

No

*if Yes - 10a. Please describe how they are assessed (mandatory free text box)

12. Please provide any other comments about LP use at your school: (non-mandatory free text box)

Section C - Barriers to Learning Plans

This section of the survey asks you to review and rate your opinion on barriers to the implementation of LPs at your medical school.

1. Please rate whether each of the following statements is a barrier to LP use at your medical school.

**Table 2:** Please rate whether each of the following statements are barriers to LP use at your medical school.

	Not a barrier	Minor barrier	Moderate barrier	Major barrier	Don’t know
Students have difficulty:					
- Creating learning goals					
- Creating specific learning goals					
- Creating realistic learning goals					
- Identifying their strengths					
- Identifying their weaknesses					
- Creating effective plans to achieve their learning goals					
- Achieving their learning goals					
- Keeping track of their progress on completing LPs					
Students:					
- Lack time to create LPs					
- Lack commitment to complete their LP					
- Feel stressed when using LPs					
- Are not engaged in the LP process					
- Lack opportunities to achieve their learning goals (ex: exposure to specific patients)					
- Do not receive feedback on their learning goal progress					
Faculty:					
- Do not guide students while creating LPs					
- Lack time to review LPs with students					
- Do not guide students while implementing LPs					
- Are not engaged with the LP process					
- Lack skills to help students with their learning goals					

2. Please provide any other comments about barriers to LP use at your school: (non-mandatory free text box to answer)

Section D - Facilitators of Learning Plans

This section of the survey asks you to review and rate your opinion on the facilitators to the implementation of LPs at your medical school.

1. Please rate whether each of the following statements is a facilitator to LP use at your medical school.

**Table 3:** Please rate whether each of the following statements are facilitators to LP use at your medical school.

	Not a facilitator	Minor facilitator	Moderate facilitator	Major facilitator	Don’t know
Students are taught:					
- About LPs					
- How to identify their strengths					
- How to identify their weaknesses					
- How to construct specific learning goals					
Students:					
- Use a framework to create their learning goals (ex: SMART)					
- Use a framework to create their LPs					
- Have protected time to create their LPs					
- Have protected time to review their LPs with faculty					
- Use a documentation system to monitor progress on their LPs					
Students receive faculty guidance to:					
- Create their learning goals					
- Create their LPs					
- Implement their LPs					
Students receive faculty feedback on their:					
- Learning goals					
- LP progress					
- LP outcomes					
Faculty are educated about:					
- LPs					
- Learning goal creation					
- Giving effective feedback specific to LP					

2. Please provide any other comments about facilitators to LP use at your school: (non-mandatory free text box to answer)

Section E - Self-Regulated Learning Strategies and Tools

This section of the survey asks you to review and rate the following statements on strategies or tools that promote SRL for students at your medical school.

1. Please select the statements that best reflect processes or tools used by your medical school.

**Table 4:** Please select the statements that best reflect processes or tools used by your medical school.

At our medical school we:	Yes	No	In development
Educate students on:			
- SRL skills			
- How to set a learning goal			
- Strategies to achieve their learning goals			
- Self-reflection skills to assess the impact of their learning			
Educate faculty on:			
- SRL skills			
- How to set a learning goal			
- Strategies to help students achieve their learning goals			
- Self-reflection skills to help students assess the impact of their learning			
- How to provide quality feedback			
Require a specific learning goal structure (ex: SMART template)			
Utilize a learning goal documentation system to monitor progress on learning goal achievement			
Schedule regular time for student creation			
Schedule regular time for students to follow up of their learning goal progress			
Require students to have a faculty mentor or advisor who assists them with learning goal creation			
Require the faculty mentor or advisor to follow up on students’ learning goal progress			

2. Please provide any other comments about the development of life-long learning skills at your medical school: (non-mandatory free text box to answer)
